# Supplementary material for: Solvent-Free Desulfurization System to Produce Low-Sulfur Diesel Using Hybrid Monovacant Keggin-Type Catalyst
Source: Molecules. 2020 Oct 27;25(21):4961. doi: 10.3390/molecules25214961 (PMC7663092; doi:10.3390/molecules25214961)
Supplement: Supplementary file 1 [file molecules-25-04961-s001.pdf]

Supplementary Information for

Solvent-Free Desulfurization System to Produce Low-Sulfur Diesel Using Hybrid Monovacant Keggin-Type Catalyst

Fátima Mirante, Baltazar de Castro, Carlos M. Granadeiro and Salete S. Balula *

LAQV-REQUIMTE, Departamento de Química e Bioquímica, Faculdade de Ciências, Universidade do Porto, 4169-007 Porto, Portugal; fatima.mirante@fc.up.pt (F.M.); bcastro@fc.up.pt (B.d.C.); cgranadeiro@fc.up.pt (C.M.G.)

* Correspondence: sbalula@fc.up.pt; Tel.: +351-220-402-576; Fax: +351-220-402-659

Academic editor: Alexander O. Terent'ev

Received: 19 September 2020; Accepted: 26 October 2020; Published: date

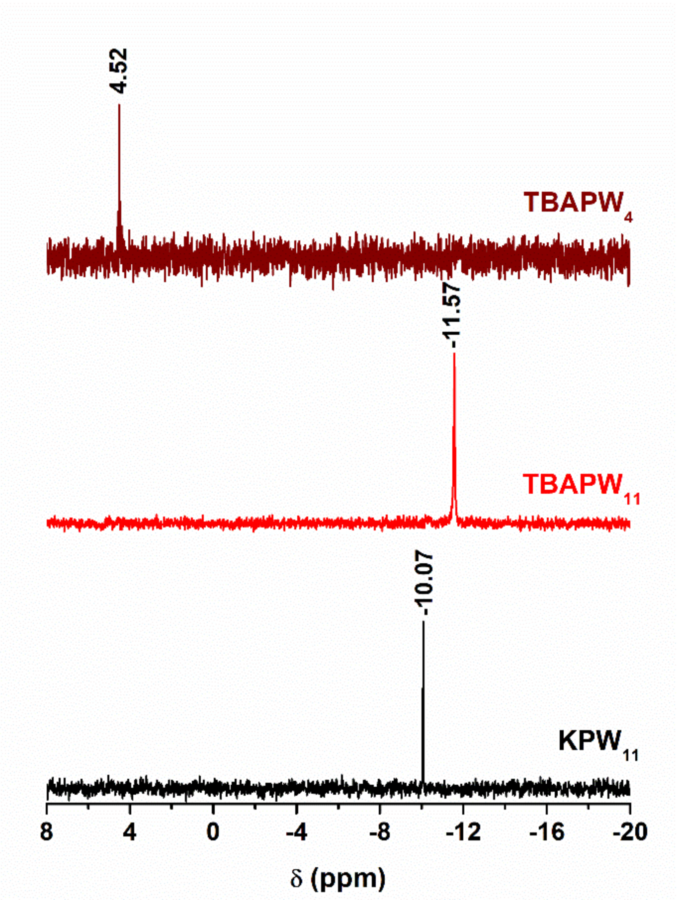

Figure 1. ^{31}P NMR spectra of the TBA[PW_4], TBA[PW_{11}] and K[PW_{11}] in CD_3CN.

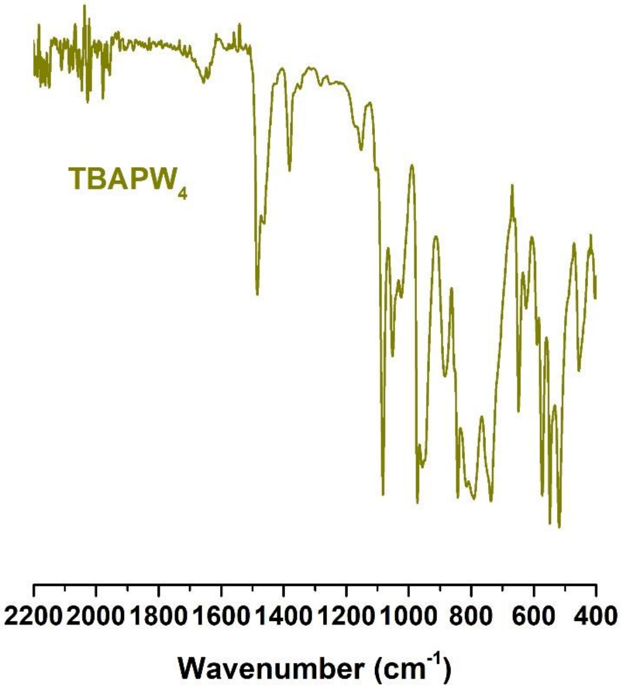

Figure 2. FTIR-ATR spectrum of TBA[PW_4].
